# Chimeric Antigen Receptor Based Therapy as a Potential Approach in Autoimmune Diseases: How Close Are We to the Treatment?

**DOI:** 10.3389/fimmu.2020.603237

**Published:** 2020-11-26

**Authors:** Muhammad Sadeqi Nezhad, Alexander Seifalian, Nader Bagheri, Sajad Yaghoubi, Mohammad Hossein Karimi, Meghdad Adbollahpour-Alitappeh

**Affiliations:** ^1^ Department of Clinical Laboratory Science, Young Researchers and Elites Club, Gorgan Branch, Islamic Azad University, Gorgan, Iran; ^2^ Blood Transfusion Research Center, High Institute for Research and Education in Transfusion Medicine, Gorgan, Iran; ^3^ Nanotechnology & Regenerative Medicine Commercialization Centre (Ltd), The London BioScience Innovation Centre, London, United Kingdom; ^4^ Cellular and Molecular Research Center, Basic Health Sciences Institute, Shahrekord University of Medical Sciences, Shahrekord, Iran; ^5^ Department of Clinical Microbiology, Iranshahr University of Medical Sciences, Iranshahr, Iran; ^6^ Transplant Research Center, Shiraz University of Medical Sciences, Shiraz, Iran; ^7^ Cellular and Molecular Biology Research Center, Larestan University of Medical Sciences, Larestan, Iran

**Keywords:** immunotherapy, autoimmune diseases, Tregs, cytotoxic T cells, CAR-T cells, CAAR-Tregs, adoptive cell therapy

## Abstract

Despite significant breakthroughs in understanding of immunological and physiological features of autoimmune diseases, there is currently no specific therapeutic option with prolonged remission. Cell-based therapy using engineered-T cells has attracted tremendous attention as a practical treatment for autoimmune diseases. Genetically modified-T cells armed with chimeric antigen receptors (CARs) attack autoreactive immune cells such as B cells or antibody-secreting plasma cells. CARs can further guide the effector and regulatory T cells (Tregs) to the autoimmune milieu to traffic, proliferate, and exert suppressive functions. The genetically modified-T cells with artificial receptors are a promising option to suppress autoimmune manifestation and autoinflammatory events. Interestingly, CAR-T cells are modified to a new chimeric auto-antibody receptor T (CAAR-T) cell. This cell, with its specific-antigen, recognizes and binds to the target autoantibodies expressing autoreactive cells and, subsequently, destroy them. Preclinical studies of CAR-T cells demonstrated satisfactory outcomes against autoimmune diseases. However, the lack of target autoantigens remains one of the pivotal problems in the field of CAR-T cells. CAR-based therapy has to pass several hurdles, including stability, durability, trafficking, safety, effectiveness, manufacturing, and persistence, to enter clinical use. The primary goal of this review was to shed light on CAR-T immunotherapy, CAAR-T cell therapy, and CAR-Treg cell therapy in patients with immune system diseases.

## Introduction

The hallmark of the immune system is its ability to distinguish self from foreign antigens (1). This capability can be misdirected against healthy tissues under certain circumstances such as breakage of immune tolerance and disrupted rearrangement of homeostasis, resulting in mistaken attack and destruction of normal host cells, known as autoimmune diseases (2). Autoimmune diseases include over 100 types of diseases, accounting for an estimated 3.1% and 7.6–9.4% cases affected in the USA and Europe, respectively (3). Based on the affected region, the age of onset, response to the therapeutic agents and clinical manifestation may vary among different people (4). Both auto-antibody-secreting B lymphocytes and self-reactive T-lymphocytes play a key role in the development of autoimmune diseases (5). Based on the extent of tissue damage, autoimmunity is classified into two general categories, including organ-specific and systemic autoimmune. The former involves a specific area of the body such as type I diabetes (T1D), multiple sclerosis (MS), rheumatoid arthritis (RA), inflammatory bowel diseases (IBDs), and myasthenia gravis (MG), while the latter affects multiple regions of the body, causing systemic lupus erythematosus (SLE) and Sjögren’s syndrome (SS) (6, 7).

A variety of mechanisms have been proposed to be involved in the development of autoimmunity. Such factors are classified into the following categories: I) epitope spreading, where infections alter the primary epitope into the secondary epitope or form several neoepitopes on antigen-presenting cells (8); II) bystander activation, also known as pre-primed autoreactive T cell activation in a T cell receptor (TCR)-independent manner (9); III) persistent virus infection, where the constant presence of viral antigens prompts immune responses (10); and IV) molecular mimicry, which is explained by immunological cross-reactivity between the host and pathogen due to shared immunologic epitopes or sequence similarities (11).

Conventional and common therapies currently used for autoimmune diseases include immunosuppressive agents, such as steroids or cytostatic drugs, analgesics, non-steroidal anti-inflammatory drugs, and glucocorticoids. Such drugs typically manage and inhibit auto-antibody production but lack the ability to completely eliminate the diseases (12, 13). The typical immunosuppressive and immunomodulatory agents, such as methotrexate, leflunomide, hydroxychloroquine, and sulfasalazine, are known as disease-modifying antirheumatic drugs (DMARDs). Each of these drugs has a particular mechanism of action that targets crucial pathways in the inflammatory cascade suppressing the immune system; however, they increase the risk of opportunistic infections (14–16). Several new drugs, known as biologic agents, have been introduced for localized treatment instead of affecting the entire immune system. These drugs include TNF-α inhibitors, belimumab and rituximab depleting B cells, T-cell co-stimulation blocker, anti-interleukin 6 (IL-6), anti-IL-1, and protein kinase inhibitors (16). In addition, monoclonal antibodies (mAbs), such as anti-TNFα, anti-CD19, anti-CD20, anti-CD22, and anti-IL6R, target multiple B cell subtypes, and other aberrant cells in autoimmune diseases (17, 18).

Immunotherapy is a field of immunology which helps immune cells fight against diseases through either enhancement or suppression and manipulation of the immune system. Biological therapy, such as immunotherapy, cytokines, cancer vaccines, and cell-based therapies with chimeric antigen receptor (CAR) T-cell, is a type of cancer treatment which augments the anti-tumor response of the host’s immune cells to fight and eliminate cancer cells (19–21).

CAR-based immunotherapy has attracted tremendous attention and is considered as a possible therapy option for autoimmune diseases. CAR modified-T cells kill aberrant immune cells such as B cells or antibody-secreting plasma cells in autoimmune diseases. CARs can guide the effector and regulatory T cells (Tregs) to the autoimmune milieu to traffic, proliferate, and exert suppressive functions (22). This study provided key features of CAR-based therapy against autoimmune diseases and critically discussed recent studies conducted on CAR modified-T cells to fight against autoimmune diseases.

### CAR Design and Key Requirements

**Figure 1:**
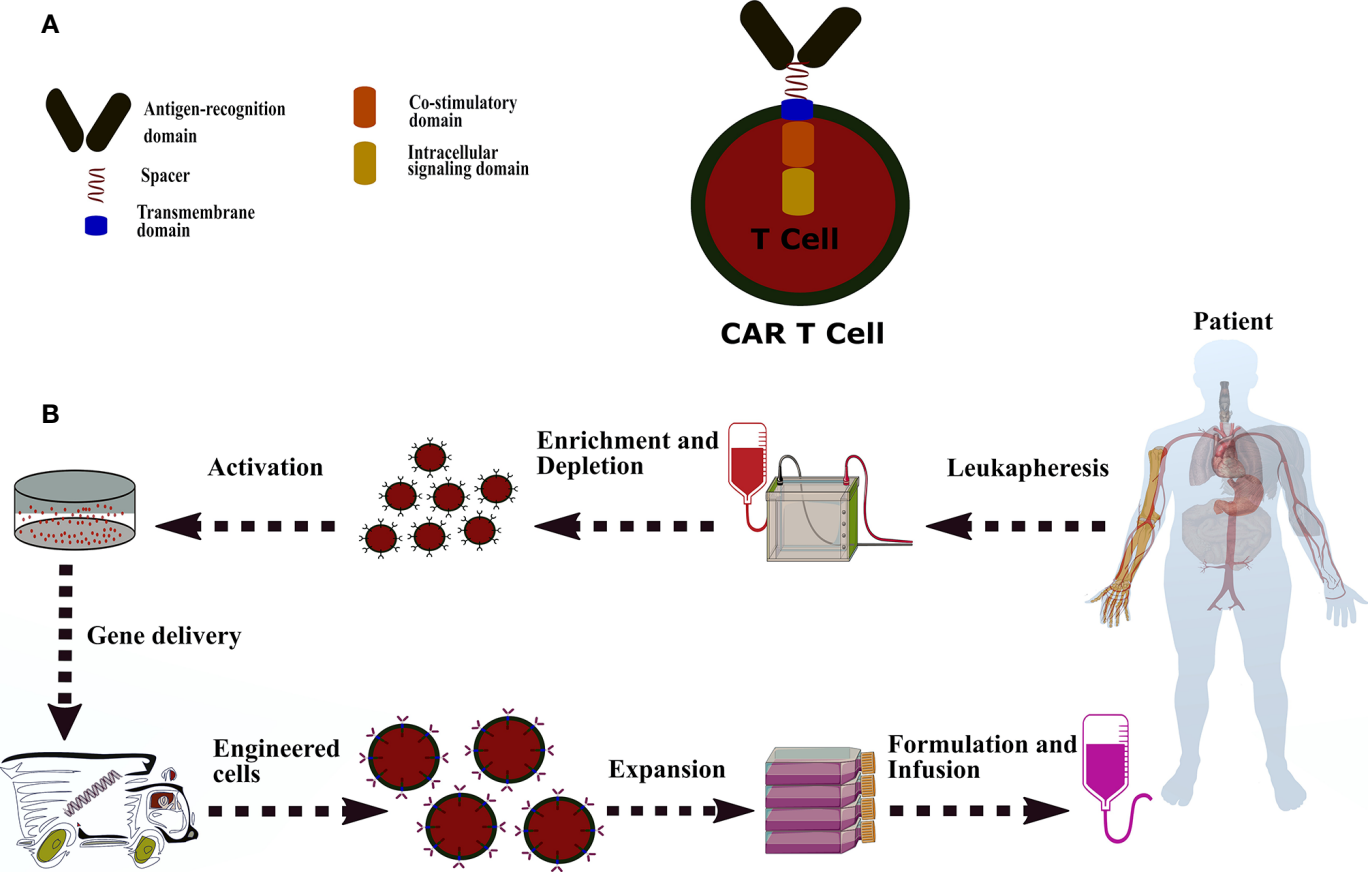
A common CAR construction. **(A)**: CAR is comprised of antigen-recognition domain (scFv), a hinge domain or spacer (CD28, CD8α, CD7, IgG4, and IgG1), a transmembrane domain (CD28, CD8α, CD7, CD4, CD3z, and OX40), a co-stimulatory domain (CD244, CD28, CD27, OX40, ICOS, and CD137), and a signaling domain (CD3z, DAP10, and DAP12). **(B)**: The process of CAR-T manufacturing from peripheral blood mononuclear cells to genetically modified-T cells and administration. CAR-T therapy starts with accumulating the patient’s white blood cells by leukapheresis. The apheresis products are enriched or deleted for a specific cell subset and then activated by one of following methods, including interleukins (IL-2, IL-7, and IL-15), anti-CD3/CD28 antibody-coated magnetic beads, soluble CD3 antibody, artificial antigen-presenting cells, plate-bound antibodies, and adhesion molecules. The activated T cells are introduced with the CAR transgene through lentiviral or retroviral and non-viral methods (electroporation of naked DNA and plasmid-based transposon/transposase). Afterwards, the engineered-T cells undergo an expansion process in static culture bags or dynamic culture vessels or rotating bioreactors. Eventually, cell numbers are calculated based on the patient’s disease burden, weight, and another formulation. The CAR modified-T cells transfer to either a container for infusion purposes or cryopreserved for storage.

CAR is a hybrid antigen receptor that redirects T cells toward cells or tissues expressing the antigen of interest and empowers the T cells to recognize antigens in a major histocompatibility complex (MHC)-independent manner (23, 24). In contrast, TCR requires peptide processing in the MHC-dependent manner to identify cells with particular human leukocyte antigen (HLA) expression (25, 26). A typical CAR is composed of three major components, including ectodomain consisting of an antigen-recognition domain and a hinge domain, a transmembrane domain, and endodomain defined in co-stimulatory(s) and an intracellular signaling domain (**Figure 1A**) (27–29).

#### Ectodomain

Ectodomain is a domain of a membrane protein outside the cytoplasm and exposed to the extracellular space, which consists of a single-chain variable fragment (scFv) and a spacer (30, 31). scFv serves as a signal peptide of ectodomain in CAR structure which is formed by a variable fragment of heavy (V_H_) and light (V_L_) chains of a mAb and fused to a flexible linker (32). Molecular engineers have continued to diversify the scFv molecule, resulting in a) bispecific scFvs that are present on two CARs separately and target two distinct antigens; b) tandem scFvs in which two scFvs expressed on one CAR construction simultaneously; and c) nano-scFvs that mostly derived from camel’s antibody (33). The antigen-recognition domain is derived mainly from variable lymphocyte receptors, TCR-mimic, and mAb. Among, scFv is designated as the most common choice of antigen-recognition domain for CAR construction (34, 35).

The hinge domain, also known as a spacer, provides a bridge-like connection between the transmembrane domain and the antigen-binding domain. The spacer gives a different range of motions to the binding domain to facilitate antigen recognition. Proteins used in the hinge region of CAR-T cells are the fragment crystallizable region (Fc region), the tail region of an antibody, of IgG1, IgG4, IgD, and cell surface molecules such as CD28, CD8α, and CD7 (36, 37).

#### Transmembrane Domain

The transmembrane domain, which consists of a hydrophobic alpha helix that spans the membrane, provides a connection between the extracellular and intracellular section of a CAR molecule. This domain also influences the expression and stability of CARs (31, 38, 39).

#### Endodomain

Despite the initial signals provided by FcR-*γ* or CD3ζ domain, full activation of T cells requires a second signal known as co-stimulation (40). The early T cells expressing the first-generation CARs (with no co-stimulatory domain) had insufficient cytokine secretion and showed disappointing outcomes *in vivo* (41, 42). Consequently, the first-generation CARs were fused with a co-stimulatory molecule to augment their proliferation and responses (43). A wide variety of co-stimulatory molecules have been investigated, including CD28, 4-1BB/CD137, OX40, ICOS, CD27, MyD88/CD40, NKG2D, CD244, and DAP10. These molecules have been, and continue to be, examined in various clinical and preclinical trials; however, among them, CD28 and 4-1BB/CD137 are the most common choices of co-stimulatories for CAR-T cell manufacturing (44–46). Upon antigen recognition, CAR endodomains transmit activation and co-stimulatory signals to the T cells. The activation relies on the phosphorylation of immunoreceptor tyrosine-based activation motifs (ITAMs) present within the cytoplasmic domain of the TCR complex and CD3-ζ domain (47, 48).

### The Principal of CAR-T Cell Manufacturing

#### T Cell Source

Because most CAR-T cells are derived from autologous T cells, the general workflow for CAR-T manufacturing begins with harvesting specific T cell subsets via leukapheresis procedure. Different commercial devices provide size-based cell fractionation for the depletion of monocytes and the isolation of lymphocytes (49). The isolated cells undergo a washing process to discard contaminated platelets or RBCs and anticoagulants. In some protocols, T cells are enriched for a specific subset of T cells, such as CD4^+^, CD8^+^, CD25^+^, and CD62L^+^ T cells. Although CD3^+^ T cells are used mainly in CAR-T clinical purposes, other subsets of T cells such as naive, central memory, and memory stem cells may also show promising advantages. T cell subsets, which provide robust or effective therapeutic attitudes and have the least side effects than other T cell populations, should be considered as a T cell source in the CAR-T cell manufacturing process. Finally, the purified T cells can be either used for the next procedure or cryopreserved for future work (50–52).

#### Activation Process

Processed T cells need sustained and adequate activation to be prepared for CAR cDNA transduction. T cells receive the first signal from their FcR-*γ* or CD3ζ domain and the second signal from co-stimulatory signals such as CD28, 4-1BB, or OX40. Technically, several activation methods are available, each of which has its own advantages and disadvantages. The methods include a) anti-CD3 based activation, in which soluble anti-CD3 mAb and IL-2 interact with T-cell surface CD3 receptors (53, 54); b) cell-based activation, in which antigen presenting cells such as dendritic cells and artificial antigen-presenting cells (K562 cell lines) are used to stimulate the expansion of CAR-T cells (55, 56); c) magnetic-bead activation, in which anti-CD3/CD28 antibody-coated magnetic beads are used for selection and *ex vivo* T-cell activation; and d) other strategies, which include plate-bound antibody, adhesion molecules (CD2), and interleukins (IL-2, IL-7, and IL-15) (57, 58).

#### CAR Transgene Delivery

Currently, CAR gene delivery relies predominantly on viral and non-viral gene transfer systems. The most popular vectors are lentiviral vectors, γ-retroviral vectors, and the transposon/transposase system. Besides, messenger RNA (mRNA)-mediated gene transfer and electroporation of naked DNA are other methods to introduce CARs into cells of interest (59).

Lentiviral vectors, as a widely used vector in CAR delivery, transduce non-dividing cells and have a high gene transfer efficiency, resulting in a safe genomic integration profile (60). In contrast, γ-retroviral vectors attracted tremendous attention as they provide multiple stable packaging cell lines with broad tropism and have a high gene expression property (61). Both of these viral vectors require complicated and expensive equipment and reagents. Therefore, a new inexpensive and straightforward gene delivery method, such as transposon/transposase system, needs to be introduced in CAR-T manufacturing platform. However, this system has a random integration and increases the possibility of oncogenic risks (62). mRNA-mediated gene transfer is another potential choice of gene delivery, as it provides a cytoplasmic gene expression and does not involve the genome of host cells. The mRNA-based gene delivery decreases genotoxicity due to its cytoplasmic expression system (63, 64).

#### Expansion Process

Several expansion procedures have been introduced for genetically modified-T cells, each of which has its unique characteristics. These systems are known as the Miltenyi CliniMACS Prodigy system, G-Rex bioreactor, GE WAVE bioreactor, and T-flask. Therefore, the expansion system should be chosen based on the CAR-T cell construction strategy (65–67).

After expansion system selection, modified-T cells are expanded into media using supplemental factors and strict control over temperature, pH, agitation, dissolved oxygen (DO) levels, gas sparging, and cytokine supplementation (68). Expansion protocols for CAR-T cells rely typically on cytokines, such as IL-2, IL-7, IL-15, and IL-21. The choice of cytokines and their concentration is likely associated with the CAR-T cell phenotypes (69–71).

Eventually, the modified-T cells are ready to be introduced into the recipient patient through IV injection or intratumoral administration (**Figure 1B**). Notably, the success of CAR-T therapy may impede due to fiasco to administer the genetically modified-T cells properly and promptly before patients reach end-stage or progressive complications.

Despite the breakthrough in CAR development, there is still no solid manufacturing process across the therapeutic platforms (49). Prior to CAR-T manufacturing, selection of a particular T cell population such as the central memory or stem cell-like memory T cells can affect the therapeutic outcomes (72–74).

### CD4 and Tregs and the Mechanism of Suppression in Autoimmune Diseases

CD4 effector T cells, also known as T helper (Th) cells, affect the immune functions by providing proper stimuli for immune cells. Classically, CD4^+^ T cells are divided into Th1 and Th2 subsets. Th1 cells express a particular transcription factor Tbet (TBX21) during viral infections to support CD8^+^ T cells. In contrast, Th2 cells are in association with humoral immune responses by assisting B cells. Th2 cells produce cytokines, such as IL-4, IL-5 and IL-13, and express transcription factor GATA3. FoxP3^+^ Tregs and IL-17 producing Th17 cells (a new subset of effector memory T cells) are dominant Th cells. Tregs play a crucial role in maintaining peripheral tolerance, while Th17 cells fight against extracellular pathogens. Factors that influence and induce the Th17 cells are IL-1β, IL-21, IL-6, and transforming growth factor-beta (TGF-β). Importantly, Th cells are believed to play a central role in human autoimmune diseases (75, 76).

Tregs are a class of T cells which participate in suppression or regulation of other cell types in the immune system and control the immune feedback to self or foreign antigens to prevent autoimmune manifestation (77). Tregs are developed from two major sources, including thymus and periphery (outside the thymus). The former produces thymic Treg cells (tTreg cells), while the latter develops induced Treg cells (iTreg cells) (78, 79). Tregs have a potent immunosuppressive function in which differentiation, development, and the suppressive manner of Tregs are closely associated with primary TCR contact (80, 81). Thymic Tregs expressing CD4, CD25, and FoxP3 are the most studied Tregs in different clinical purposes and hold promise in treating autoimmune diseases (82, 83). Under normal circumstances, Tregs have several suppressive mechanisms depending on the inflamed regions and type of immune responses. Tregs secret anti-inflammatory cytokines such as TGF-β, IL-10, and IL-35, to prevent autoimmune manifestation and autoinflammatory events (84, 85). These cytokines within the inflammation zone affect different cell subsets. After the activation, Tregs can destroy autoreactive CD4^+^ T and CD8^+^ T cells through different potential mechanisms (86). Many possible mechanisms have been proposed for suppression mechanisms used by Tregs, which include i) secreting inhibitory cytokines, such as IL-10 and TGFβ; ii) producing granzymes and perforin to induce apoptosis in target cells; iii) expressing a high level of the CD25 receptor that enables Tregs to consume more IL-2 and deplete the surrounding cells of this cytokine; iv) inhibiting the effector T-cell function by adenosine generated by CD39 and CD73 co-expression; and v) preventing the effector T-cell activation by targeting dendritic cells (6, 87, 88). There would be a particular mechanism of suppression used by Tregs that may play a central role under certain conditions to mediate immune tolerance (89). Identification of the molecular characteristics and the main suppression mechanism of Tregs enables researchers to develop promising therapeutic approaches such as CAR-Tregs to fight against autoimmune diseases.

### CD8^+^ T Cells and the Mechanism of Suppression in Autoimmune Diseases

T cells stem from common lymphoid progenitor cells in the bone marrow and migrate through the bloodstream to the thymus. They undergo a series of maturation stages to express some specific cell surface receptors, such as TCR, CD4, and CD8 receptors (90, 91). Among, cytotoxic T cells, also known as CD8^+^ T cells or cytotoxic lymphocytes (CTLs), are characterized based on their cytolytic activities. Naïve CD8 T cells circulate through secondary lymphoid organs where they are activated via interaction between their TCR and antigen-presenting cells, such as dendritic cells. After activation, the characteristics of CD8 cells are determined through changes in their functions, gene expression, and migration. They begin to differentiate and proliferate to turn to cytotoxic T cells (CTLs). These cells detect infected or malignant cells by direct contact or in a TCR-dependent manner, destroy the target cells and provide a safe harbor for other cells. The mechanism used by CTLs to eliminate the infected cells lie within different strategies, including secretion of chemokines, perforin, granzymes, expression of Fas ligand, and effector cytokines such as tumor necrosis factor and interferon-γ mechanism (92, 93).

CTLs fight autoimmune diseases by exerting different strategies against infected cells. They suppress B cells through a CD40 pathway to mitigate the CD23^+^ cells and secretion of IL-10. Meanwhile, they influence the balance between Th1 and Th2 population, by decreased Th2 population (94, 95). In inflammatory conditions, CTLs recognize and destroy oligodendrocytes, astrocytes, and neurons/axons expressing MHC-I molecules by secreting perforin, IFN-γ, and TNF-α (96). Nevertheless, CTLs can produce some specific cytokines such as IL-10, IL-4, and TGF-β that negatively regulate the CD4^+^ T cell’s performance and proliferation (97–99). This characteristic highlights the regulatory role of CTLs on CD4^+^ T cells in autoimmune conditions. Despite the present evidence that shows the cytotoxic and regulatory role of CTLs in autoimmune conditions, the exact mechanism of CTLs in autoimmune diseases has not been understood profoundly and requires comprehensive investigations.

### CAR-Treg Therapy in Autoimmune Diseases

Autoimmune diseases are defined by a loss of tolerance. Cells with immunosuppressive attitudes like Tregs play a significant role in restoring the immune system (100). Strategies to genetically manipulate Tregs are a promising option to suppress the autoimmune manifestation and the autoinflammatory events (**Table 1**) (104).

**Table 1:** CAR-Treg-based therapy in preclinical models of autoimmune diseases.

Condition	Target antigen	CAR-construct	Delivery route	Preclinical study	Dosage of CAR-Tregs	Overall outcomes	Year [Ref]
**Type 1 diabetes**	Insulin	CAR-Treg cells (insulin scFv, CD28, CD3ζ)	Retroviral	BALB/cJ, C57BL/6J, and non-obese diabetic (NOD/LtJ) mic	25×10^5^	The study showed successful treatment of CAR-Tregs without affecting the general immune competence of the recipient.	2019 (101)
**Ulcerative colitis**	Carcinoembryonic antigen (CEA)	CEA CAR-Tregs (SCA431 scFv, CD28, CD3ζ)	Retroviral	CEABAC-2 and CEABAC-mice (n=10)	15–30 × 10^5^	In general, CAR-Tregs were successfully tested in two distinct complementary model systems, indicating the viability of CAR-Treg-based treatment.	2014 (102)
**Multiple sclerosis**	Myelin oligodendrocyte glycoprotein	MOG CAR-Tregs (MOG scFv, CD28, CD3ζ	Lentiviral	Female C57BL/6 mice	1 × 10^5^	CAR-Tregs targeting myelin localized to the CNS efficiently suppressed ongoing inflammation and alleviated disease symptoms.	2012 (103)

*Tenspolde et al.* used adoptive immunotherapy with genetically engineered-Tregs expressing an insulin-specific CAR against NOD/LtJ female mice with type 1 diabetes (T1D). Because there are a small number of Tregs specific for diabetes-associated antigens in patients, the generation of CAR-modified Tregs would encounter a problem due to the lack of Tregs of interest. Therefore, they converted CD4^+^ effector T cells into Tregs by introducing the Foxp3 gene. Further, effector T cells were transduced with gamma-retroviral encoding second-generation insulin-specific CAR plasmid, providing converted insulin CAR-modified Treg cells (insulin CAR-cTregs). Their findings revealed that infusion of 2.5×10^6^ of insulin CAR-cTregs was ineffectual to prevent NOD/Ltj female mice from becoming diabetes. However, insulin CAR-cTregs persisted approximately 4 months in diabetic mice. The possible explanation for this phenomenon can be ascribed to storage and biological form or molecular structure of insulin. There might be other forms of insulin in the body that hinder the treatment process, such as a hexamer (a unit of six insulin molecules) and a monomer as an active form. The diversity of insulin structure is expected to hinder the therapeutic effect of engineered-T cells; therefore, a strategy to neutralize other forms of insulin could enhance the success of CAR-T cells. Insulin CAR-cTregs were also shown to have minimal off-target toxicity due to their high specificity to insulin (101).

**Figure 2:**
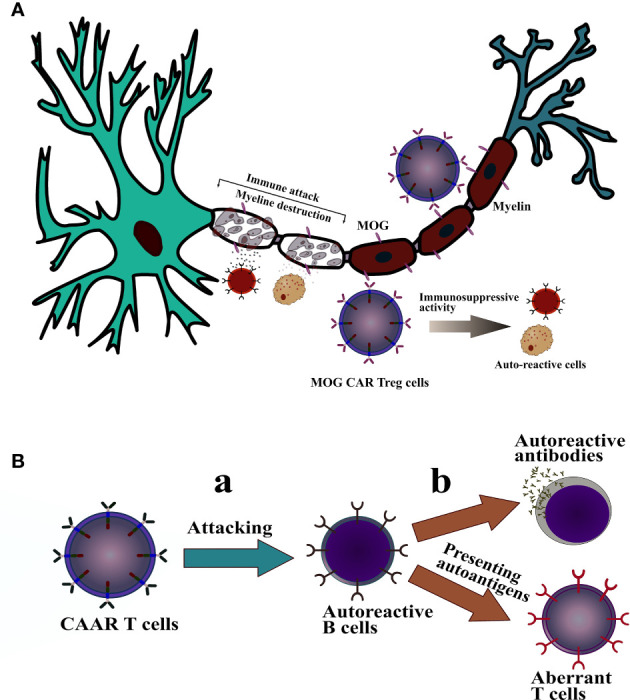
The mechanism of action of CAAR-T cells and CAR-Treg cells against autoimmune disease. **(A):** MOG CAR-Treg cells effectively bind and is localized to the MOG^+^ oligodendrocytes in CNS and exert their immunosuppressive manner to protect myelin against immune attack. **(B)**: (a); T cell expressing CAAR specific for B cell receptors targets aberrant B cells. (b); further, it inhibits B cell development from secreting autoreactive antibodies and prevents B cells from presenting autoantigens to autoreactive T cells, which leads to suppression of T cell-mediated autoimmune diseases.

Likewise, a study transduced CD4^+^ T cells using a lentiviral vector system encoding the myelin oligodendrocyte glycoprotein (MOG) CAR gene and Foxp3 gene. The main role of MOG CAR-Tregs is to localize and bind to MOG^+^ oligodendrocytes in CNS, providing a protective shield for these cells against immune attacks (**Figure 2A**). In this study, 1 × 10^5^ of MOG CAR-Tregs infused through intraperitoneal delivery routes in female C57BL/6 mice afflicted with encephalomyelitis (EAE). Their findings demonstrated that MOG engineered-Tregs could treat all the EAE mice, decrease the IL-12 and IFN levels, and suppress effector T cell proliferation 10 days after infusion. The treated mice were again introduced with the second dose of EAE-inducing inoculum and, as a consequence, the MOG CAR-Tregs effectively protected the MOG^+^ cells against EAE inflammation. Fifteen days post cell treatment, mice were analyzed using the immunohistochemical technique. The results revealed reactive astrogliosis and myelination of axons in mouse brains treated with MOG CAR-Tregs. In contrast, the number of CAR-Tregs decreased during migration to the target site where MOG^+^ cells are present. This low number may interpret into different concepts, including on-target/off-tumor toxicity, insufficient trafficking and migration, and immunogenicity (103). Thus, further evaluation is required to study the probability of these phenomena in MOG CAR-Treg treatment.

Furthermore, CEA transgenic CAR was introduced into Tregs to generate CEA CAR-Tregs for preventing ulcerative colitis (UC) development, a form of inflammatory bowel disease. CEA CAR-Tregs were able to suppress the occurrence of colitis and decreased the manifestation of colitis-associated colorectal cancer. CEA CAR-modified Tregs trafficked and expanded in the site of inflammation, just for 7 days, and finally were removed by day 9. Findings highlighted that the low persistence of engineered-Tregs might be associated with immunogenicity due to the presence of CEA CAR antibodies in the sera of treated mice (102).

Recently, *Boroughs et al*. have investigated the types of co-stimulation domains used in CAR-Tregs to identify the best co-stimulatory serving in persistence, phenotype maintenance, survival, and proliferation in engineered-Tregs. They engineered primary human Tregs with the second-generation CAR containing either CD28 or 4-1BB co-stimulatory domain, and then monitored the effects of each domain in a preclinical model and *in vitro*. Their results demonstrated that the CD28 domain was more beneficial in CAR-Treg’s suppressive behavior and maintained Treg phenotypes compared with 4-1BB co-stimulatory domain. CAR-Tregs with the CD28 domain were also capable of suppressing the effector T cell–mediated graft rejection and secreting more IL-10. Of note, the 4-1BB signaling domain attenuated the immunosuppressive activity of CAR-Tregs, whereas CD28-based CAR-Tregs expressed a high level of LAP and CTLA4 and depleted more of IL-2 in the zone of interest (105). Hence, using CD28 as a co-stimulatory domain in CAR construction outperforms the 4-1BB co-stimulatory domain.

### CAR-T Therapy in Autoimmune Diseases

Rituximab, a mAb targeting the B-lymphocyte restricted surface antigen CD20, is a promising therapeutic approach against some autoimmune diseases, especially B cells (106–108). Adoptive cell therapy with CD19 CAR-T cells demonstrated significant success in hematologic malignancies (109). The application of CAR-based therapy can be drawn into autoimmune diseases to eliminate malignant B cells producing autoantibodies.

Researchers developed CD19 CAR engineered-T cells to eliminate aberrant CD19^+^ B cells in two lupus mouse models. CD19 CAR-T cells could successfully eliminate CD19^+^ B cells, leading to decreased auto-antibody secretion. Additionally, the disease burden was ameliorated in both female NZB/W and MRL-lpr mice. The serology of modified T cell-treated mice exhibited decreased levels of total IgM and IgG antibodies as well as Anti-DNA IgG and IgM. However, all the IgM^+^ B cells were not removed completely, and plasma IgM was detectable in mice. Further success was detected in the pathogenesis and survival of mice. The life span of both treated mice increased to almost one year, demonstrating the profound effect of CAR-based therapy in autoimmune diseases. This long persistence of CD19 CAR-T cells in mice led to a functional test to assess whether modified-T cells have cytotoxic efficacy toward B cells. Results indicated that CD19 CAR-T cells actively continue to eliminate B cells up to 11 months and CD19^+^ B cell aplasia has occurred during the treatment. Moreover, RNA analysis highlighted that modified-T cells did not eliminate normal B cells whether splenic B cell populations or plasma cells resident in the bone marrow. These findings clearly suggested that CD19 CAR-T cells could remain functional for months and remove transferred autologous CD19^+^ B cells without harming other B cell populations in the bone marrow of murine lupus (110).

Recently, a novel study has redirected genetically modified-T cells expressing scFv of mAb287 against A^g7^ I-B:9–23(R3) complex on antigen-presenting cells (APCs) to assess the therapeutic effects of CAR-T cells in a NOD mouse model of T1D. The B:9–23 peptide is defined by amino acids 9 to 23 of the insulin B chain which binds to MHC class II molecules of APCs. The modified-T cells were accumulated in pancreatic lymph nodes by 9 and 14 days after infusion, highlighting the favored homing and migration of the CAR-T cells where APCs present their cognate antigens. Five-week-old female NOD mice were introduced to these CAR-T cells and then examined for T1D development and CAR protective attitudes. Findings revealed that none of the treated mice developed diabetes before 18 weeks of age until only 35% of them were diabetes-free by 25 weeks. Autoantibodies to insulin (IAA) were measured to determine the CAR-T suppressive manner against autoantibodies development. Evidence indicated no IAAs at 4 weeks post-infusion, but IAAs were detected in 5/17 treated mice at 14 weeks, meaning that CAR-T cells could inhibit islet autoimmunity. Meanwhile, pancreatic lymph nodes were analyzed at 25 weeks of age to determine the persistence of modified-T cells. No engineered-T cells were detected in the treated mice, showing a limited lifespan of CAR-T cells. Taken together, these CAR-modified T cells could effectively kill APCs presenting I-Ag7:B:R3 complexes, signifying that APCs expressing the pathogenic T cell epitope are associated with autoimmunity and can be prevented by genetically modified-T cells (111). **Table 2** shows further comprehensive details of CAR T-based treatment in the studies mentioned above.

**Table 2:** CAR-T-based therapy in preclinical models of autoimmune diseases.

Condition	Target antigen	CAR-construct	Delivery Route	Preclinical Study	Dosage of CAR-T cells	Overall Outcomes	Year [Ref]
**Type 1 diabetes**	I-A^g7^-B:9–23(R3) complex	CD8+ CAR-T cells. (mAb287 scFv,4-1BB, CD28, CD3ζ)	Retroviral	Female NOD/LtJ, Thy1.1 NOD, and NOD.SCID mice	5 ×10^6^	CAR-T cells can selectively target pathogenic MHC class II: peptide complex relevant to an autoimmune disorder. The study only delayed type 1 diabetes mice and did not prevent the development of T1D.	2019 (111)
**Pemphigus vulgaris**	Keratinocyte adhesion protein Dsg3	Dsg3 CAAR-T (Dsg3, CD137-CD3ζ)	NA	Mice bearing human skin xenografts, NSG (NOD-scid-gamma) immunodeficient mouse	NA	CAAR-T cells demonstrated a targeted-based therapy against antibody-mediated autoimmune diseases with the potential generation of long-term memory.	2016 (112)
**Systemic lupus erythematosus**	CD19	CD19 CAR-T cells (CD19 scFv, CD28 and two intracellular signaling domains, CD28, CD3ζ	Retroviral	Female NZB/W or MRL-lpr mice	1.2×10^6^	CD19^+^ B cells depletion led to inhibition of auto-antibody production, and mitigated the manifestations of lupus pathogenesis, and increased the lifespans of mice	2019 (110)

Chimeric auto-antibody receptor T (CAAR-T) cells are the modified form of CAR-T cells which identify cells secreting antibodies like autoreactive B cells. The construction of CAAR-T cells consists of a specific antigen, a transmembrane domain, and an intracellular signaling domain with or without a co-stimulatory domain. CAAR-T cells recognize and bind to the target autoantibodies expressed on autoreactive cells via the specific antigen, and subsequently, destroy them. Importantly, one of the key considerations before constructing CAAR-T cells is the specific antigen of CAAR. This section should meticulously be designed to develop a specifically engineered epitope to recognize cognate autoantibodies (14). The concept of targeting pathogenic autoimmune cells is an alternative approach for autoimmune diseases. *Ellebrecht et al.* conducted a study that armed T lymphocytes with desmoglein (Dsg) 3 CAAR to target cells expressing anti-Dsg3 B cell receptors (BCRs), which are responsible for pemphigus vulgaris (PV) (**Figure 2B**). The preclinical-based experiments showed that Dsg3 modified CAAR-T cells remarkably reduced Dsg3 serum antibody levels and controlled the PV hybridoma growth. Dsg3 CAAR-T cells further destroyed the anti-Dsg3 BCR^+^ selectively and spared the BCR^–^ cells even in the presence of soluble serum anti-Dsg3 IgG. This attitude indicates that circulating autoantibodies do not prevent CAAR-T cells activity and efficacy. Next, Dsg3 CAAR-T cells were exposed to keratinocytes expressing desmocollins and desmogleins to assess their off-target activity. They showed no cytotoxicity upon encountering the keratinocytes. Furthermore, Dsg3 CAAR-T cells were introduced to human skin-xenografted mice and exhibited no significant cytotoxicity or off-target activity, highlighting their low risks of general immunosuppression. Finally, the study emphasized the applicability of targeted therapy of antibody-mediated autoimmune diseases by CAAR-T cells as a promising therapeutic option. A further consideration is required to evaluate the long-term side effects of modified-T cells and possible mutations in corresponding epitopes (112).

### Clinical Perspective: Challenges and Barriers

To the best of our knowledge, there are less than 10 clinical trials, registered at http://ClinicalTrials.gov, using CAR-T cells for the treatment of autoimmune diseases. Autoimmune diseases are caused by physiologic immune responses to self-antigens. Several distinct autoantigens have been discovered in different autoimmune diseases. Treatment can easily be developed for diseases where the culprit target antigens are identified. The concept of autoantigen-based immunotherapy, especially using CAR-T cells, has brought a state-of-the-art therapeutic approach for autoimmune diseases. CAR-T development is easier when the target autoantigens are known. Researchers have analyzed the desired autoantigens to shed light on their characteristics and physiological features. It is well-understood that the power of CAR-T cells defines by their antigen-recognition domain. This domain is mostly derived from mAb; therefore, developing a specific mAb against one of these autoantigens can enhance the specificity and success of CAR-T cell therapy in autoimmune diseases. CAR-T cells with a specific antigen-recognition domain avoid unexpected toxicities and spare the healthy tissues. Furthermore, one of the key components of CAAR-T cells is the antigen specific. The exact construction and characteristic of target autoantigens boost the therapeutic success of CAAR-T cells because these engineered-T cells recognize cells secreting antibodies like autoreactive B cells. Here, some of the potential autoantigens have been identified and classified to help future CAR or CAAR T cells design.

**Type 1 diabetes**: carboxypeptidase H, chromogranin A, glutamate decarboxylase, Imogen-38, insulin, insulinoma antigen-2 and 2β, islet-specific glucose-6-phosphatase catalytic subunit related protein (IGRP), and proinsulin (113).

**Rheumatoid arthritis:** citrullinated protein, collagen II, heat shock proteins, and human cartilage glycoprotein (114).

**Systemic lupus erythematosus:** double-stranded DNA, La antigen, nucleosomal histones and ribonucleoproteins (snRNP), phospholipid-β-2 glycoprotein I complex poly (ADP-ribose) polymerase, and Sm antigens of U-1 small ribonucleoprotein complex (115).

**Multiple sclerosis**: α enolase, aquaporin-4, β-arrestin, myelin basic protein, myelin oligodendrocytic glycoprotein, and proteolipid protein S100-β (116, 117).

**Other conditions:** celiac disease (R1-type reticulin), pernicious anemia (gastric H^+^/K^+^-ATPase), and adrenalitis (21-hydroxylase, 17-hydroxylase, and the cytochrome P450 side-chain cleavage enzyme) (118).

Genetically modified-T cells demonstrated a promising therapeutic approach against antibody-mediated autoimmune diseases and potentially eliminated autoreactive cells with no significant off-target toxicities (112). Despite several preclinical studies conducted in autoimmune diseases which resulted in satisfying outcomes, CAR-based therapy has to pass several hurdles, including stability, durability, trafficking, safety, effectiveness, manufacturing, and persistence to enter the realm of clinical use. Another pivotal concern is the dosing strategy since the primary goal of CAR-based therapy is to be curative with low-cost and time-efficient. In contrast to hematological malignancies, few studies of CAR T-based therapies have been conducted in autoimmune diseases. Therefore, obstacles and challenges are somehow unclear when it comes to CAR-T cell therapy against autoimmune diseases. Nevertheless, some of the hurdles of hematological CAR-T cell therapies seem to be closely associated with obstacles in autoimmune CAR-T cell therapy. Importantly, the lack of exclusive antigens hinders the establishment of highly specific engineered-T cells. CAR-T cells with low specificity can result in severe off-target effects and preclude the clinical application. Therefore, the first step is to identify pivotal autoantigens in autoimmune diseases. Other hurdles that CAR-T cells may encounter are insufficient homing of engineered-T cells or inadequate numbers of CAR-T cells into the inflamed zone where they cannot destroy and fight against infected cells properly. One constructive approach is to equip CAR-T cells with appropriate chemokine receptors to respond to chemokines from the inflamed area. Furthermore, factors that may limit the success of CAR-T cells against autoimmune diseases include i) the lack of an exclusive antigen in most of the autoimmune diseases; ii) inefficient trafficking of CAR-T cells to the inflamed site; iii) insufficient *ex vivo* expansion of CAR-T cells to proliferate and persist; iv) off-target effects as a result of the heterogeneous expression of the targeted antigens; v) action of immunosuppressive cells against CAR-T cells; vi) the lack of supplementary and growth factors, and vii) toxicities resulting from cytokine release syndrome (mostly by pro-inflammatory cytokines such as TNF-*α*, IL-6, IFN-*γ*, and IL-2) and neurotoxicity (119, 120).

### CAR-Based Therapies: Future Perspective

Current conventional and common autoimmune disease therapies are based on anti-inflammatory and immunosuppressive agents, including steroids or cytostatic drugs, and engineered biologics such as humanized mAbs. The constant introduction of such agents leads to some pernicious side effects and increases the chance of infections (121). A novel practical therapeutic strategy, CAR-based therapy, demonstrated a promising treatment option for leukemia and lymphoma patients. The same approach against solid tumors was ineffectual and laborious due to various identified and unidentified physiological factors (122). Indeed, the application of CAR-based therapy has not been investigated thoroughly in autoimmune diseases because there is no appropriate antigens to target them on the aberrant autoreactive cells selectively. By the advent of next-generation sequencing (NGS), different types of epitopes can be predicted and identified for future CAR-based immunotherapy (123). In addition, genome editing technology, CRISPR/Cas9, has a great influence on detecting hidden self-antigens in autoimmune diseases to increase the specificity of CAR-T cells (124). Nevertheless, the success of CAR-T cells profoundly relies on the scFv of the extracellular domain. The scFv is widely derived from murine mAb. This type of scFv increases the concern of immunogenicity; however, the problem can be handled using a humanized version of scFv (125).

In terms of using CAR modified-Tregs, several key features should meticulously be considered. Tregs have a potent immunosuppressive function in different autoimmune diseases. A mutation in their transcription factor Foxp3 can influence the suppressive manner of Tregs and lead them to severe autoimmune conditions. Tregs encounter different cellular milieu and inflammation zones. Within each zone, Tregs alter their functions and undergo the apoptosis process, signifying the feeble stability of Tregs because they can convert their immunosuppressive manner into an effector function (126). Meanwhile, the low rate of Tregs in peripheral blood is the other challenge for high scale production; thus, a strategy to increase the number of Tregs is imperative for clinical purposes. One distinguished strategy to produce Tregs is to introduce the Foxp3 gene into CD4^+^ effector T cells.

Interestingly, natural killer cells have also shown an alternative source of T cells for adoptive cell therapy. Studies demonstrated that La/SSB CAAR NK92MI cells were successfully redirected to La/SSB-specific B cell receptor-bearing lymphoma cells, a model of antibody-mediated autoimmune diseases, *in vitro* (127). Other approaches, such as pre-treatment of cells with some specific agents that elevate the expression of target antigens, may address some of the hurdles. Using other therapeutic strategies, such as immune checkpoint antibodies, combination treatments, and tyrosine kinase inhibitors, may represent interesting results. Approaches to identify several candidate antigens of targets and managing the associated-hurdles of CAR-based therapy should be prioritized and measured. Based on the types of autoimmune diseases, selecting the appropriate cells among different potential sources such as T cells, Tregs, and NK cells for engineering may increase the therapeutic success. Thus, it needs to consider the physiological aspects of these cells. These include the biology, co-receptor ligation, cytokines and chemokines, and the potential side effects or cytotoxicity of the cells to boost the therapeutic outcomes.

## Conclusion

By the advent of CAR-T cell therapy, autoimmune diseases have entered a new era of therapy. CAR-T cells can be considered as a promising alternative option for conventional based treatment due to their fewer side-effects compared to current drug therapy. However, more preclinical studies, possibly under GLP (good laboratory practice) are required for evaluation of CAR-based treatment before embarking on multicentral clinical trials. Since there are not many studies dealing with CAR-T cell therapy against autoimmune diseases, these pioneer studies mentioned earlier brought the concept of cell-based treatment in autoimmune diseases and paved the way to demonstrate that a viable novel therapy is on its way to clinical use.

## Author Contributions

All authors contributed to the study conception and design. Data acquisition: MSN, and AS. Quality control of data and algorithms: NB, SY, and MHK. Data analysis and interpretation: MSN, MK, MA-A, and NB. Manuscript preparation: MSN, AS, and SY. Manuscript editing: MSN, MHK, and MA-A. Manuscript review: MSN, AS, MHK, and MA-A. All authors contributed to the article and approved the submitted version.

## Conflict of Interest

AS was employed by The London BioScience Innovation Centre.

The authors declare that the research was conducted in the absence of any commercial or financial relationships that could be construed as a potential conflict of interest.
